# Mice with mutations in *Trpm1*, a gene in the locus of 15q13.3 microdeletion syndrome, display pronounced hyperactivity and decreased anxiety-like behavior

**DOI:** 10.1186/s13041-021-00749-y

**Published:** 2021-03-30

**Authors:** Tesshu Hori, Shohei Ikuta, Satoko Hattori, Keizo Takao, Tsuyoshi Miyakawa, Chieko Koike

**Affiliations:** 1 Graduate School of Pharmacy, Ritsumeikan University, Kusatsu, Shiga Japan; 2 Laboratory for Systems Neuroscience and Developmental Biology, College of Pharmaceutical Sciences, Ritsumeikan University, Kusatsu, Shiga Japan; 3 Graduate School of Life Sciences, Ritsumeikan University, Kusatsu, Shiga, Japan; 4 Center for Systems Vision Science, Research Organization of Science and Technology, Ritsumeikan University, Kusatsu, Shiga Japan; 5 Ritsumeikan Global Innovation Research Organization (R-GIRO), Ritsumeikan University, Kusatsu, Shiga Japan; 6 Institute for Comprehensive Medical Science, Fujita Health University, Toyoake, Aichi Japan; 7 Department of Behavioral Physiology, Faculty of Medicine, University of Toyama, Toyama, Toyama Japan; 8 Research Center for Idling Brain Science, University of Toyama, Toyama, Toyama Japan; 9 Center for Genetic Analysis of Behavior, National Institute for Physiological Sciences, Okazaki, Aichi Japan

**Keywords:** 15q13.3 microdeletion syndrome, TRPM1, Hyperactivity, ADHD, Visual impairment, Retinal ON bipolar

## Abstract

The 15q13.3 microdeletion syndrome is a genetic disorder characterized by a wide spectrum of psychiatric disorders that is caused by the deletion of a region containing 7 genes on chromosome 15 (*MTMR10*, *FAN1*, *TRPM1*, *MIR211*, *KLF13*, *OTUD7A*, and *CHRNA7*). The contribution of each gene in this syndrome has been studied using mutant mouse models, but no single mouse model recapitulates the whole spectrum of human 15q13.3 microdeletion syndrome. The behavior of *Trpm1*^−/−^ mice has not been investigated in relation to 15q13.3 microdeletion syndrome due to the visual impairment in these mice, which may confound the results of behavioral tests involving vision. We were able to perform a comprehensive behavioral test battery using *Trpm1* null mutant mice to investigate the role of *Trpm1*, which is thought to be expressed solely in the retina, in the central nervous system and to examine the relationship between TRPM1 and 15q13.3 microdeletion syndrome. Our data demonstrate that *Trpm1*^−/−^ mice exhibit abnormal behaviors that may explain some phenotypes of 15q13.3 microdeletion syndrome, including reduced anxiety-like behavior, abnormal social interaction, attenuated fear memory, and the most prominent phenotype of *Trpm1* mutant mice, hyperactivity. While the ON visual transduction pathway is impaired in *Trpm1*^−/−^ mice, we did not detect compensatory high sensitivities for other sensory modalities. The pathway for visual impairment is the same between *Trpm1*^−/−^ mice and *mGluR6*^−/−^ mice, but hyperlocomotor activity has not been reported in *mGluR6*^−/−^ mice. These data suggest that the phenotype of *Trpm1*^−/−^ mice extends beyond that expected from visual impairment alone. Here, we provide the first evidence associating TRPM1 with impairment of cognitive function similar to that observed in phenotypes of 15q13.3 microdeletion syndrome.

**Supplementary Information:**

The online version contains supplementary material available at 10.1186/s13041-021-00749-y.

## Introduction

TRPM1, the first member of the melanoma-related transient receptor potential (TRPM) subfamily to be discovered, is the visual transduction channel downstream of metabotropic glutamate receptor 6 (mGluR6) in retinal ON bipolar cells (BCs) [1, 2]. Humans with an autosomal recessive form of complete congenital stationary night blindness show mutations in TRPM1 and *Trpm1* mutant mice exhibit the lack of a b-wave in electroretinograms and the absence of light responses in ON BCs﻿ [3]. *TRPM1* is located in human chromosome 15q13.3, a region associated with 15q13.3 microdeletion syndrome, which is a genetic disorder caused by the deletion of a ~1.5 megabase region from break-point 4 to break-point 5, comprising 7 genes: *MTMR10*; *FAN1*; *TRPM1*; *MIR211*; *KLF13*; *OTUD7A*; and *CHRNA7* (OMIM #612001) [4]. The prevalence of 15q13.3 microdeletion syndrome is estimated to be 0.02% in otherwise healthy individuals [5]. Although most individuals are heterozygous, those who are homozygous have impaired vision [6–10].

Individuals with 15q13.3 microdeletion syndrome may present with mild to moderate intellectual disability, mild learning delays, autism spectrum disorder, epilepsy (recurring seizures), attention-deficit/hyperactivity disorder (ADHD), schizophrenia, bipolar disorder, and visual impairment [7, 11, 12]. The phenotype of 15q13.3 microdeletion syndrome is complex and heterogeneous [12, 13]; the prevalence of developmental delay or intellectual disability in these patients is higher than 80%, whereas that of hyperactivity or attention deficit disorder is approximately 10% to 20%.

In humans, deletion of *CHRNA7* is thought to account for the neuropsychiatric disorders in 15q13.3 microdeletion syndrome, but the phenotype of *Chrna7-*deficient mice does not recapitulate the human phenotype of this syndrome [12]. *Otud7a* mutant mice exhibit many of the same features, as patients with 15q13.3 microdeletion syndrome, including neurological features, reduced body weight, developmental delay, abnormal electroencephalogram patterns and seizures, reduced ultrasonic vocalizations, decreased grip strength, impaired motor learning/motor coordination, and reduced acoustic startle [14].

The role of TRPM1 in behavioral disorders has not been studied, probably in part because of its strong relationship with vision. TRPM1 and its regulator, mGluR6, cause congenital stationary night blindness. In TRPM1 and mGluR6 mutant retinas, the ON but not the OFF visual pathway fails to respond to light stimuli [1, 15, 16]. We previously reported an unexpected difference between *Trpm1*^−/−^ and *mGluR6*^−/−^ mouse retinas. By recording spiking in retinal ganglion cells (RGCs) using a multielectrode array, we observed spontaneous oscillations in *Trpm1*^−/−^ retinas, but not *mGluR6*^−/−^ retinas [17]. We also previously reported that rod ON BC terminals were significantly smaller in *Trpm1*^−/−^ retinas than in *mGluR6*^−/−^ retinas [17]. These data indicate that a deficiency of TRPM1, but not of mGluR6, in rod ON BCs may affect synaptic terminal maturation and underlie the observed differences in the oscillatory response. Prompted by our observation of *Trpm1*-deletion specific RGC oscillations and the location of the gene in the targeted region of 15q13.3 microdeletion syndrome, we searched for central and behavioral changes that might contribute to a persistent, rhythmic visual outflow.

In the present study, we thoroughly examined *Trpm1*^−/−^ mice by testing them in a battery of behavioral tests [18]. We further investigated structural and functional changes in *Trpm1*^−/−^ mouse brain that could potentially explain the abnormal behaviors exhibited by this mutant mouse strain as a model of 15q13.3 microdeletion syndrome.

## Methods

### Animals and Experimental Design

*Trpm1*^−/−^ mice were generated as described previously [1]. In this study, we analyzed *Trpm1*^−/−^ mice and their wild type (WT) littermates on the 129 Sv/Ev Taconic background. All behavioral tests were performed using male mice 11 to 12 weeks of age at the start of the testing (*Trpm1*^−/−^ mice, *n* = 24; WT littermates, *n* = 24). Mice were housed as pairs of *Trpm1*^−/−^ and WT mice (2 pairs/cage) with a 12-h light/dark cycle (light on from 7:00 AM to 7:00 PM). All mice had access to food and water ad libitum. Behavioral testing was performed between 8:30 AM and 6:30 PM, unless otherwise noted. Table 1 shows the behavioral test battery. After the tests, all the testing apparatuses were cleaned with diluted hypochlorous solution or 70% ethanol to prevent a bias due to olfactory cues.

**Table 1:** Comprehensive behavioral test battery in *Trpm1*^−/−^ mice

Test	Age (weeks old)
General health/neurological screen	11–12
Grip strength/wire hang test	11–12
Light/dark transition (Dark box start)	11–12
Open field	11–12
Elevated plus maze	12–13
Hot plate	12–13
Social interaction (novel environment)	12–13
Rotarod	12–13
Social approach and novelty preference (Crawley’s ver.)	13–14
Acoustic startle response and prepulse inhibition	13–14
Porsolt forced swim	15–16
Gait analysis	16–17
Barnes maze_Training	20–23
Barnes maze_PT1(24 h)	22–23
Barnes maze_PT2 (1 M)	27–28
T-maze spontaneous alternation	31–32
Light/dark transition (Light box start)	34–35
Tail suspension	34–35
Contextual and cued fear conditioning_Day1 (conditioning)	34–35
Contextual and cued fear conditioning_Day2 (context and cued)	34–35
Contextual and cued fear conditioning_Day30 (remote memory)	38–39
Wire hang test_2nd	38–39
Social interaction in home cage	39–40
Home cage test (daily activity)	40–43
Open field test + MPH (10 mg/kg)	74–75
Open field test + MPH (3 mg/kg)	79–81

Brain weight measurement and monoamine quantification in brain tissues were performed with 129 Sv/Ev male at 4 months (*Trpm1*^−/−^ mice, *n* = 24; WT littermates, *n* = 24) or 1 month (*Trpm1*^−/−^ mice, *n* = 4; WT littermates, *n* = 5). Gene expression analysis was performed with 129 Sv/Ev male mice at 1 month (WT, *n* = 5). Mice used for monoamine quantification were housed in pairs of *Trpm1*^−/−^ mice and WT mice (2 pairs/cage) with a 12-h light/dark cycle (light on from 8:00 AM to 8:00 PM), and tissue dissection was performed at the same time-point (1:00 PM). All mice had access to food and water ad libitum*.* The experimental procedures and housing conditions for animals were approved by Institutional Animal Care and Use Committee of National Institute for Physiological Sciences, Fujita Health University and Ritsumeikan University.

### General health and neurological screening

A general health and neurological screen to evaluate the body weight, rectal temperature, whisker and coat condition, as well as simple reflexes such as righting, whisker touch, eye blink, ear twitch reflexes and reaching behavior as described previously [19]. A grip strength test and wire hang test were conducted to measure muscle strength. Grip strength was measured using a grip strength meter (O’Hara & Co., Japan). In the wire hang test, the mouse was placed on a wire cage lid that was then inverted so that the subject gripped the wire. Latency to fall onto the bedding was recorded, with a 60-s cutoff time.

### Light/dark transition test

The light/dark transition test was performed as described previously [20–22]. The apparatus used for the light/dark transition test consisted of a cage (21 × 41.5 × 25 cm) divided into 2 sections of equal size by a partition with a door (O’Hara & Co., Japan). One section was brightly illuminated (390 ± 20 lux), whereas the other section was dark (<2 lux). Mice were placed into the dark side of the apparatus and allowed to move freely between the 2 sections for 10 min with the door open. In the same way, mice 34–35 weeks of age were placed into the light side of the apparatus and allowed to move freely between the 2 sections for 10 min. The total number of transitions, time spent in each section, initial latency to enter the light section, and distance traveled were recorded automatically using Image LD software.

### Open field test

The open field test was performed as described previously [21, 22]. Mice were allowed to move freely in an open field apparatus (40 × 40 × 30 cm; Accuscan Instruments, USA) illuminated at 10.0 lux for 120 min. Each subject was placed individually into the corner of the apparatus. The total distance, vertical activity (rearing measured by counting the number of photobeam interruptions), time spent in the center area, and stereotypic behaviors were recorded.

### Elevated plus maze test

The elevated plus maze test was performed as described previously [21, 23]. The apparatus (O’Hara & Co., Japan) consisted of 2 open arms (25 × 5 cm) and 2 enclosed arms of the same size, with a central square (5 × 5 cm). The enclosed arms were surrounded by 16-cm high transparent walls. To minimize the likelihood of an animal falling from the apparatus, 3-mm-high Plexiglas ledges were provided for the open arms. The arms were made of white plastic plates elevated to a height of 50 cm above the floor. Arms of the same type were arranged at opposite sides to each other. Mice were placed in the central square of the maze, facing one of the enclosed arms and behavior was recorded during a 10-min test period. The percentage of open arm entries, percentage of time spent on the open arms, total number of arm entries, and total distance traveled were measured automatically using Image EP software.

### Hot plate test

The hot plate test was performed as described previously [23]. Mice were placed on a 55.0 °C hot plate (Columbus Instruments, USA), and latency to the first hind paw response, either a foot shake or paw lick, was recorded.

### Social interaction test

The social interaction test was performed as described previously [16]. A pair of mice (12–13 weeks old) was placed simultaneously at opposite corners in the open field apparatus (40 × 40 × 30 cm; O’Hara & Co., Japan), whose illumination level was 10.0 lux at the center of the floor, and allowed to explore freely for 10 min. Each mouse had been housed in different cages. The number of active contacts, number of contacts, mean duration per contact, total duration of contact, and total distance traveled were measured. The analysis was performed automatically using Image SI software.

### Rota-rod test

Motor coordination and balance were tested with the rota-rod test old as described previously [23]. The rota-rod test using an accelerating rota-rod (UGO Basile, Italy) was performed by placing a mouse on a rotating drum (3 cm diameter) and measuring the time each animal was able to maintain its balance on the rod. The speed of the rota-rod accelerated from 4 to 40 rpm over a 5-min period.

### Social approach and novelty preference test

Social approach and preference for social novelty were tested with the 3-chamber social test apparatus as described previously [21, 23]. The apparatus comprised a rectangular, 3-chambered box and a lid with a video camera (O’Hara& Co., Japan). Each chamber was 20 cm × 40 cm × 22 cm and the dividing walls had small openings (5 cm × 3 cm) to allow exploration into each chamber. The day before testing, the mice were individually placed in the middle chamber and allowed to freely explore the entire apparatus for 10 min. During the test session, the amount of time spent in each chamber and the time spent around each cage were recorded and analyzed automatically using Image CSI.

### Acoustic startle response/prepulse inhibition tests

The acoustic startle response/prepulse inhibition tests were performed as described previously [23] (O’Hara & Co., Japan). A test session began by placing a mouse in a Plexiglas cylinder where it was left undisturbed for 10 min. The duration of white noise that was used as the startle stimulus was 40 ms for all trial types. A test session consisted of 6 trial types (i.e., 2 types for startle stimulus-only trials and 4 types for prepulse inhibition trials). The intensity of the startle stimulus was 110 or 120 dB. The prepulse with an intensity of 74 or 78 dB was presented 10.0 ms before the startle stimulus. Four combinations of prepulse and startle stimuli were used (74 –110, 78 –110, 74 –120, and 78–120). Six blocks of the 6 trial types were presented in pseudorandom order such that each trial type was presented once within a block. The average intertrial interval was 15 s (range, 10–20 s).

### Porsolt forced swimming test

Depression-related behavior was assessed using the forced swimming test as described previously [19]. The apparatus consisted of a Plexiglas cylinder (22 cm height × 12 cm diameter). The cylinder was filled with water (room temperature, 23 ± 2 °C) to a height of 7.5 cm. Mice were placed into the water, and their behavior was recorded over a 10-min test period. Immobility and distance traveled were analyzed automatically using Image PS software.

### Gait analysis

The gait during walk/trot locomotion was assessed using DigiGait Imaging System (Mouse Specifics, USA) as described previously [24]. Digital video images of the underside of mice were collected at 150 frames/s. We placed the mice on a treadmill belt moving at a speed of 24.7 cm/s. The percent time of the stride or stance duration, stride length, stance width, step angle, and paw angle were measured.

### Barnes maze

The Barnes maze test was performed as described previously [19]. The circular open field (O’Hara & Co., Japan) was elevated 97 cm from the floor. From 1 to 3 training sessions were conducted each day. At 24 h after the 15th training session, a probe test was conducted without the escape box to confirm that this spatial task was acquired based on navigation by distal environmental room cues. One month after the last (16th) training session, probe trial tests were conducted again to evaluate memory retention. After 5 additional training sessions conducted after the memory retention test, the escape box was moved to a new position opposite to the original (reversal learning). Mice were then trained with 8 sessions to locate the new position of the escape hole using the same procedure as described above. Latency to reach the target hole, distance to reach the target hole, number of errors and time spent around each hole were recorded automatically using Image BM software.

### T-maze spontaneous alternation

The T-maze spontaneous alternation test was performed as described previously [22] using an automatic modified T-maze apparatus (O’Hara & Co., Japan). Mice were subjected to the spontaneous alternation protocol for 5 sessions. One session consists of 10 choices with a 50-min cutoff time. Mice were first placed in the start compartment of the T-Maze. Mice chose entering either the left or the right arm and could return to the start compartment. The mice were then given a 3-s delay followed by a free choice between both T arms. A correct choice was made if the mouse entered the arm that was not visited in the previous choice. The percentage of correct responses, latency (s) to complete a session, and distance traveled during the session were measured. Data acquisition was performed automatically using Image TM software.

### Tail suspension test

Depression-related behavior was assessed by the tail suspension test as described previously [24]. Mice were suspended 30 cm above the floor in a visually isolated area by adhesive tape placed 1 cm from the base of the tail, and their behavior was recorded over a 10-min test period. Data acquisition and analysis were performed automatically using Image TS software.

### Contextual and cued fear conditioning

The ability of mice to learn and remember an association between environmental cues and aversive experiences was assessed by fear conditioning test as described previously [22, 23]. Each mouse was placed in a test chamber (26 × 34 × 33 cm, O’Hara & Co., Japan) and allowed to explore freely for 2 min. A 55-dB white noise, which served as the conditioned stimulus (CS), was presented for 30 s. Next, a mild (2 s, 0.3 mA) foot shock, which served as the unconditioned stimulus (US), was presented immediately after the CS. Two more CS-US pairings were presented with a 2-min interstimulus interval. Context testing was conducted 1 day after conditioning in the same chamber for 30.0 s without CS and US presentations.

Cued testing with altered context was conducted after conditioning using a triangular box (33 × 33 × 33 cm) made of white opaque Plexiglas, which was located in a different room. Mice were allowed to explore the chamber for 360 s. In the first 3 min, neither a CS nor US was presented, then a CS (a 55 dB white noise) was presented for the last 3 min. Freezing and distance traveled were recorded. Data acquisition, control of stimuli (i.e. tones and shocks), and data analysis were performed automatically using Image FZ software.

### The 24-h home cage monitoring test

The 24-h home cage test was performed as described previously [22]. The system for monitoring social interaction comprised a home cage (19 × 29 × 13 cm) and a filtered cage top with an infrared video camera (O’Hara & Co., Japan). Two mice with the same genotype that had been housed separately were placed together in a home cage. To evaluate their locomotor activity and social interaction, their behavior was monitored with a video camera for 1 week. ﻿Distance traveled was measured automatically using ImageHA software. The occurrence of a social interaction was detected by counting the number of particles consisting of the mice as follows: 2 particles indicated that the mice were not in contact with each other whereas 1 particle indicated that 2 mice were in contact with each other. The locomotor activity of the mice was also measured.

### Methylphenidate administration in the open field

After the behavioral test battery, the behavioral response to methylphenidate (MPH) was assessed in the open field. A quarter of the area of the open field apparatus (20 × 20 × 30 cm) was used by installing a divider. Other conditions were the same as for the open field test. Mice of each genotype were randomly divided into 2 groups for treatment with MPH and saline. The experiment was repeated twice with varying drug doses (3 mg/kg or 10 mg/kg). Locomotor activity was recorded continuously during the 60-min habituation period and for 120 min after injection of saline or MPH.

### Monoamine quantification in brain tissues

Monoamine transmitter quantification was performed as described previously [25]. Tissue concentrations of biogenic monoamines were analyzed after dissection of various brain regions, including the prefrontal cortex, hippocampus, striatum, cerebral cortex, olfactory bulb, cerebellum, midbrain, pons and medulla, thalamus,  and hypothalamus. The weight of the brain tissue was measured and homogenized in 0.2 M ice-cold perchloric acid (including 10.0 µM EDTA 2Na) and the homogenates were deproteinated by cooling on ice for 30 min. The homogenates were centrifuged at 20,000 g for 15 min at 0 °C. Then, the pH of the supernatant was adjusted to approximately 3.0 by adding 1 M sodium acetate. The samples were filtered through a 0.45-mm filter (Millipore, Billerica, USA). Next, 10 µL of the filtrate was loaded into a high performance liquid chromatography (HPLC) system (Eicom, Japan). The HPLC system had a ø3.0 mm × 150 mm octadecyl silane column (SC-5ODS, Eicom, Japan) and an electrochemical detector (ECD; HTEC-50.0; Eicom, Japan) set to an applied potential of + 750 mV versus an Ag/AgCl reference analytical electrode. The change in electric current (nA) at 25 °C was recorded using a computer interface. The mobile phase was composed of 0.1 M aceto-citric acid buffer (pH 3.5), methanol, sodium-1-octane sulfonate (0.46 M), and EDTA 2Na (0.015 mM) [830: 170: 1.9: 1]. The flow rate was 0.5 mL/min.

### Gene expression analysis in the brain

Total RNA was isolated from each brain part using Biomasher II (NIPPON GENE, Japan) and ISOGEN II (Nippi, Japan). For complementary DNA synthesis, 1 µg of total RNA was reverse-transcribed (RT) into complementary DNA using the SuperScriptIII (TaKaRa, Japan) according to the manufacturer’s instructions. Quantitative polymerase chain reaction (qPCR) was conducted on a Thermal Cycler Dice® Real Time System II (TaKaRa, Japan) using TB Green® *Premix Ex Taq*™ II (Tli RNaseH Plus) (TaKaRa, Japan) according to the manufacturer’s instructions. Primers used for mouse *Trpm1*: forward, 5′-GAGATGCAGCCCAAACTGAAGC-3′; reverse, 5′-TGACGACACCAGTGCTCACA-3′. Primers for mouse b-*actin*: forward, 5′- CTCTGGCTCCTAGCACCATGAAGA -3′; reverse, 5′- GTAAAACGCAGCTCAGTAACAGTCCG -3’.

### Corticosterone measurement

Blood was collected from mice at 4 months of age by cardiac puncture immediately after cervical dislocation. The serum was separated by centrifuging at 2,000 g for 20 min, and stored at -80℃ until use. Serum corticosterone measurements were performed by enzyme-linked immunosorbent assay (ELISA) using a Corticosterone immunoassay (R&D Systems, USA) according to the manufacturer’s instructions.

### Image analysis

Behavioral data were obtained automatically by customized applications based on a public domain ImageJ program (Image LD, Image EP, Image SI, Image CSI, Image PS, Image BM, Image TM, Image TS, Image FZ, Image HA). The ImageJ plugins, and the precompiled plugins for light/dark transition test (Image LD), elevated plus maze (Image EP), open field test (Image OF), fear conditioning test (Image FZ), and T‐maze (Image TM) are freely available on the website of “Mouse Phenotype Database” (http://www.mouse-phenotype.org/software.html).

### Data analysis

All statistical analyses were performed using Graph Pad Prism7. Statistical methods are indicated in the figure legends. Data are presented as mean ± SEM. An unpaired 2-tailed Student’s *t* test or Welch’s *t* test were used for 2-group comparisons. A 2-way analysis of variance (ANOVA) or repeated-measures 2-way ANOVA followed by Tukey’s test or  a 1-way ANOVA followed by Dunnett’s test was used for multiple comparisons. Unless otherwise noted, the *p* values are for the genotype effect.

### Data repository

The raw data of the behavioral tests and the information about each mouse are accessible on the public database “Mouse Phenotype Database” (http://www.mouse-phenotype.org/).

## Results

### *Trpm1*^−/−^ mice show significantly high daily locomotor activity

**Fig. 1:**
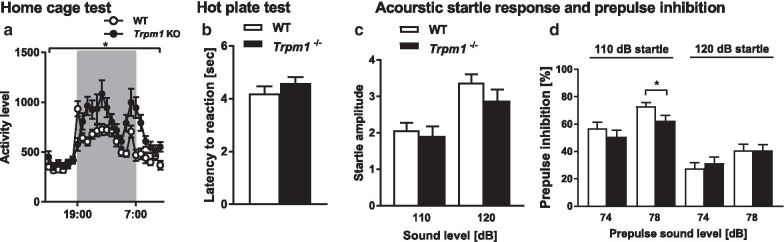
Physical characteristics of *Trpm1*^−/−^ mice. **a** Daily activity averaging 6 days in the home cage; *n* = 22 for both genotypes. **b** Latency to the first response in the hot plate test. *n* = 24 for both genotypes. **c**, **d** Acoustic startle response/prepulse inhibition tests; **c** amplitude of the startle response to the 110 and 120 dB acoustic stimuli, **d** percentage of prepulse inhibition at the 74 and 78 dB prepulse sound level. *n* = 24 for both genotypes. *P < 0.05; repeated measures 2-way ANOVA (**a**), Student’s *t* test (**b-d**)

We performed a battery of more than 20 behavioral tests (Table 1). There was almost no significant difference for general physical characteristics, such as body weight, body temperature, grip strength, and motor coordination between *Trpm1*^−/−^ and WT mice (Additional file 1: Fig. S1A–K). *Trpm1*^−/−^ mice showed no depression-like behaviors in the Porsolt forced swim test and tail suspension test (Additional file 1: Fig. S1L–N). Intriguingly, *Trpm1*^−/−^ mice showed significantly high daily locomotor activity (Fig. 1a).

We examined sensory responses in *Trpm1*^−/−^ mice, but found no significant difference between *Trpm1*^−/−^ mice and WT mice in the hot plate test, acoustic startle response, or prepulse inhibition (Fig. 1b–d).

### Hyperactivity and reduced anxiety-like behavior in *Trpm1*^−/−^ mice

**Fig. 2:**
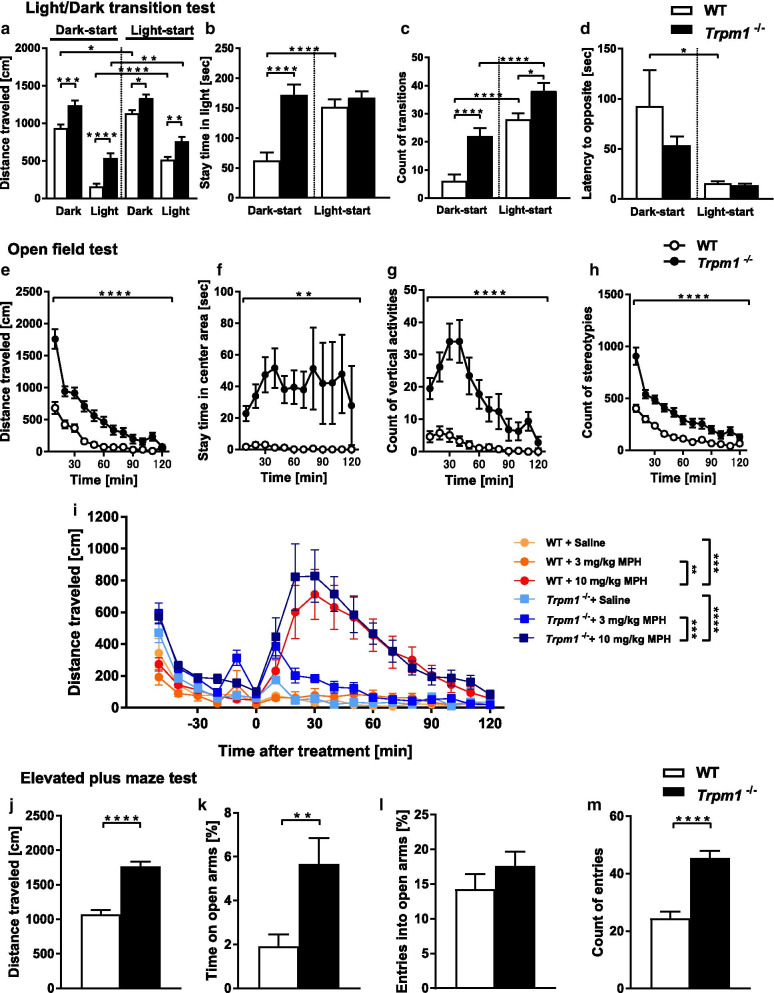
Locomotor activity and anxiety-like behavior of *Trpm1*^−/−^ mice. Light/Dark transition test; **a** total distance traveled, **b** time spent in light, **c** number of transitions, **d** latency of opposite side. *n* = 24 for both genotypes. Open field test; **e** total distance traveled, **f** time spent in center of the field, **g** number of vertical activities, **h** number of stereotypies. *n* = 24 for both genotypes. **i** Total distance traveled with treatment of MPH. *n* = 9 for WT + Saline, *n* = 9 for WT + 3 mg/mL MPH, *n* = 12 for WT + 10 mg/mL MPH, *n* = 11 for *Trpm1*^−/−^  + saline, *n* = 12 for *Trpm1*^−/−^  + 3 mg/mL MPH, *n* = 12 for *Trpm1*^−/−^  + 10 mg/mL MPH. Elevated plus maze test; **j** total distance traveled, **k** time spent on open arms, **l** number of entries into open arms, **m** and number of entries. *n* = 24 for both genotypes. *P < 0.05, **P < 0.01, ***P < 0.001, ****P < 0.0001; 3-way ANOVA followed by Tukey’s multi comparison test (**a–d**), repeated measures 2-way ANOVA (**e–h**), repeated measures 2-way ANOVA followed by Tukey’s multi comparison test (**i**), Student’s *t* test (**j**, **l**, **m**), Welch’s *t* test (**k**)

To assess anxiety-like behavior, we performed the light/dark transition test, open-field test, and elevated plus maze test (Fig. 2). In the light/dark transition test, distance traveled in the light and dark chamber was significantly increased in *Trpm1*^−/−^ mice,  suggesting reduced anxiety-like behavior (Fig. 2a). The defect in the ON visual pathway may promote a longer stay time in the light, and increased transition time and shorter latency to light for tests started at dark (Fig. 2b–d). In the open field test, which measures voluntary locomotor activity in a novel environment, *Trpm1*^−/−^ mice exhibited a significant increase in total distance, vertical activity, center time, and stereotypic behavior relative to WT mice (Fig. 2e–h), suggesting strong hyperactivity, which also explains the longer distance traveled in the light/dark transition test. To investigate hyperactivity in *Trpm1*^−/−^ mice with ADHD, we performed the open field test after administering MPH (Fig. 2i) [26]. At 120 min after administering the MPH, both WT and *Trpm1*^−/−^ mice showed prominent hyperactivity, especially mice that  were injected with 10 mg/kg MPH. These findings does not support the idea that the ADHD-like behavior displayed in *Trpm1*^−/−^ mice can be reduced by MPH administration [27].

Additionally, in the elevated plus maze test, *Trpm1*^−/−^ mice exhibited a significantly increased number of entries and longer traveled distance compared with WT mice, behaviors that are also explained by hyperactivity (Fig. 2j, m). Although visually impaired, *Trpm1*^−/−^ mice did not show differences in entries to open arms, but stayed a longer time in open arms, suggesting reduced anxiety-like behavior (Fig. 2k, l).

To examine the cause of the reduced anxiety-like behavior in *Trpm1*^−/−^ mice, we examined serum corticosterone levels in *Trpm1*^−/−^ mice by ELISA. A reduction in anxiety should correlat with a decrease in serum corticosterone levels [28, 29], as a reduction in anxiety-like behavior in the absence of a decrease in serum corticosterone levels may have some other cause. The serum levels of corticosterone were not significantly different between *Trpm1*^−/−^ mice and WT mice (Additional file 1: Fig. S1O).

### Abnormal social interaction in *Trpm1*^−/−^ mice

**Fig. 3:**
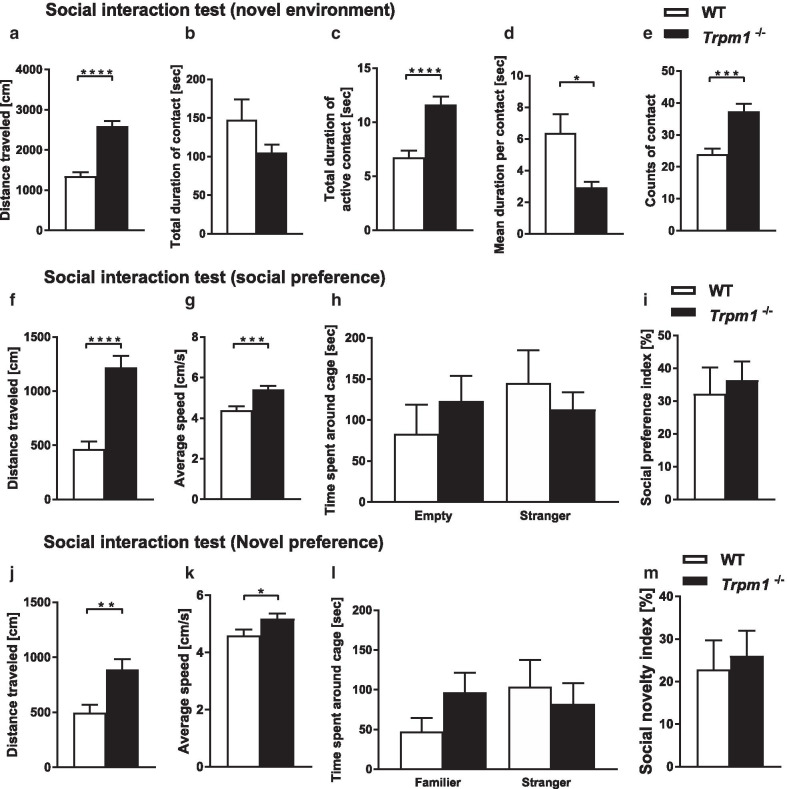
Social interaction of *Trpm1*^−/−^ mice. Social interaction in a novel environment; **a** total distance traveled, **b** total duration of contact, **c** total duration of active contact, **d** number of contacts, **e** mean duration per contact. *n* = 12 for both genotypes. Social preference; **f** total distance traveled, **g** average speed, **h** time spent in each chamber, **i** social preference (calculated as the ratio of time spent in stranger chamber to all chamber). *n* = 24 for both genotypes. Social novel preference; **j** total distance traveled, **k** average speed, **l** time spent in each chamber, **m** novel preference (calculated as the ratio of time spent in stranger chamber to all chambers; *n* = 24 for both genotypes. *P < 0.05, **P < 0.01, *** P < 0.001, **** *P* < 0.0001; Student’s *t* test (**a**, **c**, **e–g**, **h**; left, **i**, **j–m**), Welch’s *t* test (**b**, **d**, **h**; right)

Four kinds of social interaction tests (novel environment, sociability, novelty preference, and home cage test) were performed to evaluate social behaviors in *Trpm1*^−/−^ mice (Fig. 3). The novel environment test revealed significant differences between *Trpm1*^−/−^ and WT mice, including a shorter duration per contact, increased contact number, and total distance traveled, which may be explained by the hyperactivity of *Trpm1*^−/−^ mice (Fig. 3a, d, e). Although the total duration of contact tend to be shortened, active contacts by *Trpm1*^−/−^ mice had a longer duration (Fig. 3b, c). Neither Crawley’s sociability and social novelty preference test nor the test in the home cage revealed significant differences between WT and mutant mice (Fig. 3f–m, Additional file 1: Figure S1P).

### Attenuation of fear and spatial memories in *Trpm1*^−/−^ mice

**Fig. 4:**
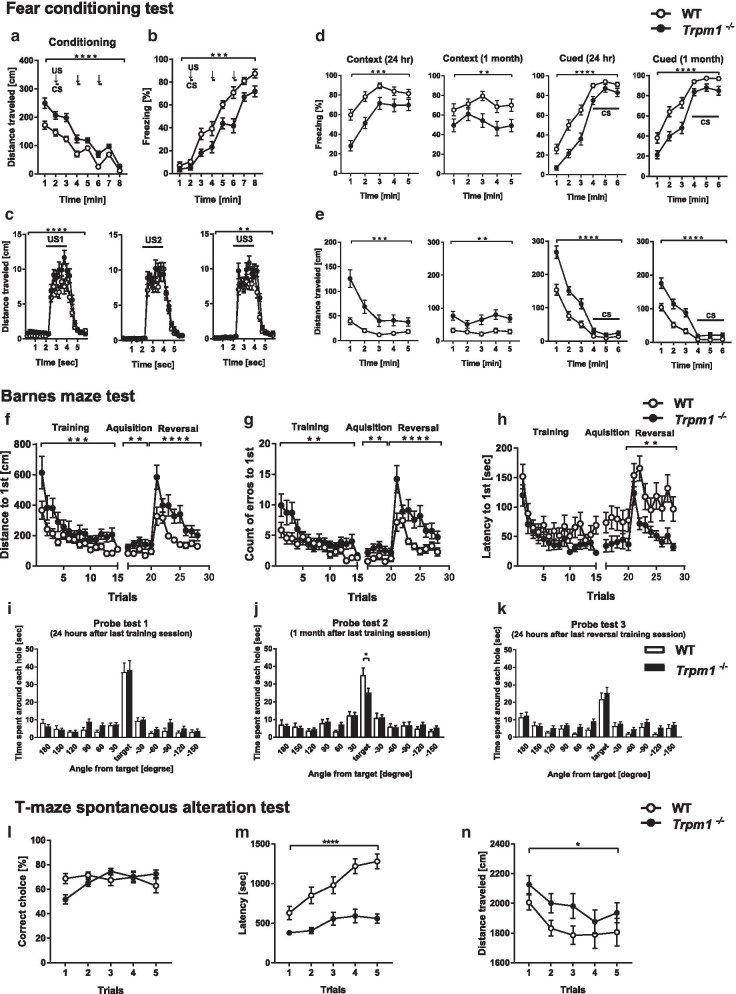
Cognitive function of *Trpm1*^−/−^ mice. Fear conditioning test; **a** distance traveled in the conditioning phase, **b** percentage of freezing time in the conditioning phase. Conditioned stimulus (CS: white noise) and unconditioned stimulus (US: foot shock) were presented, **c** distance traveled during and after foot shocks, **d** percentage of freezing time in the context tests or cued tests at 1 day and 30 days after conditioning, **e** distance traveled in the context tests or cued tests at 1 day and 30 days after conditioning. *n* = 24 for both genotypes. Barnes maze test; **f** distance, **g** error count, **h** latency to first reach the correct hole above the escape box in the training, acquisition and reversal sessions, Time spent around each hole in the probe trial conducted 24 h (**i**), 1 month (**j**) after the last training session and 24 h after last reversal training session (**k**). *n* = 24 for both genotypes. T-maze forced alternation task test; **l** percentage of correct responses, **m** latency, **n** distance traveled. *n* = 24 for both genotypes. *P < 0.05, **P < 0.01, ***P < 0.001, ****P < 0.0001; repeated measures 2-way ANOVA (**a–h**, **l–n**), Student’s *t* test (**i**, **k**), Welch’s *t* test (**j**)

The contextual and cued fear conditioning test is used to assess fear memory (Fig. 4). In the conditioning phase, *Trpm1*^−/−^ mice showed a lower level of freezing and traveled longer distances during sessions (Fig. 4a, b). The mutant mice traveled longer immediately after foot shock, an index of pain sensitivity (Fig. 4c). At 24 h after conditioning, *Trpm1*^−/−^ mice showed decreased freezing and increased distance traveled. Similar significant differences were observed in tests 28 days after conditioning (Fig. 4d, e).

We performed the Barnes maze test to determine whether the fear memory deficit in  *Trpm1*^−/−^ mice contributes to hyperlocomotion or results from a deficit of memory. In both training sessions and reversal task tests, the distance to the escape box (Fig. 4f) and the number of errors to reach the escape box were significantly higher in *Trpm1*^−/−^ mice (Fig. 4g), but the latency to first reach the escape box was equivalent or shorter in *Trpm1*^−/−^ mice than in WT mice (Fig. 4h), which may be related to hyperlocomotor activity. The probe tests were performed at 24 h and 1 month after the final training sessions. In these tests, *Trpm1*^−/−^ and WT mice exhibited a significant effect of target hole location against the other holes: 24 h, WT *p* < 0.0001, *Trpm1*^−/−^ *p* < 0.0001; 1 month, WT *p* < 0.0001, *Trpm1*^−/−^ *p* < 0.0001; 1-way ANOVA followed by Dunnett’s multiple comparison test), indicating that both genotypes were able to distinguish the location of the target. Time spent around the correct hole did not differ significantly between genotypes at 24 h after training, but was significantly shorter in *Trpm1*^−/−^ mice 1 month later (Fig. 4i, j). These results suggest that *Trpm1*^−/−^ mice have a deficit in long-term memory. In the reversal probe test, although both genotypes distinguished the location of the target (WT *p* < 0.0001, *Trpm1*^−/−^ *p* < 0.0001; 1-way ANOVA followed by Dunnett’s multiple comparison test), there was no significant difference in time spent around the correct hole between both genotypes (Fig. 4k). This result indicates that *Trpm1*^−/−^ mice have no deficit in behavioral flexibility. We also performed a T-maze test to examine working memory in *Trpm1*^−/−^ mice. Although *Trpm1*^−/−^ mice had a significantly shorter latency and a significantly longer distance traveled, the number of correct responses at each trial was not significantly different from that in WT mice (Fig. 4l–n). Taken together, *Trpm1*^−/−^ mice showed attenuated fear and long term memory, but no obvious deficit in flexibility and working memory.

### Abnormal structural and biochemical changes in the brains of *Trpm1*^−/−^ mice

**Fig. 5:**
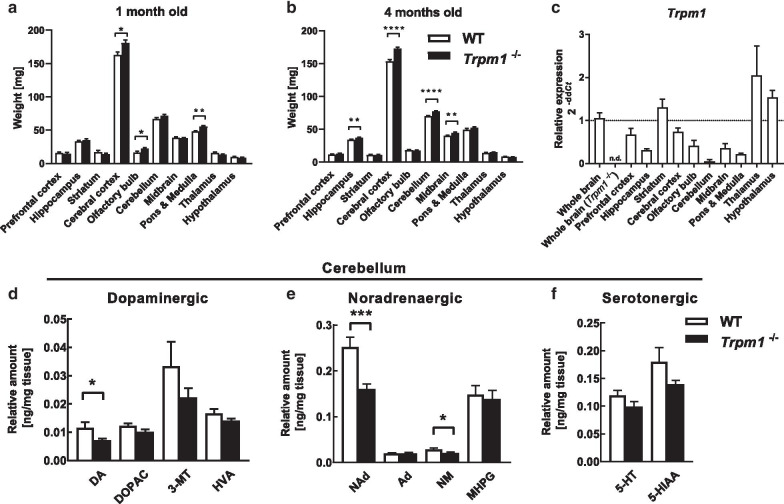
Structural and biochemical abnormalities in *Trpm1* ^–/–^ mice. Brain weight at 1 month old (**a**), *n* = 5 WT, 4 *Trpm1*^–/–^ and 4 months old (**b**), *n* = 24 for both genotypes. **c** Expression of *Trpm1* gene in WT brain. *n* = 4 for both genotypes. All amplification data were normalized with mean cycle threshold (*Ct*) value of WT whole brain group for Δ*Ct* and normalized with β-actin for Δ*Ct*. *Trpm1* mRNA was not detectable in whole brain of *Trpm1*^–/–^ mice. Quantification of monoamine neurotransmitters in the cerebellum at 4 months old; **d** dopaminergic, **e** noradrenergic, **f** serotonergic neurotransmitters, and their metabolites. DOPAC: 3,4-dihydroxyphenylacetic acid, 3-MT: 3-methoxytyramine, HVA: homovanillic acid, Ad: adrenaline, NM: normetanephrine, MHPG: 3-methoxy-4-hydroxyphenylglycol, 5-HIAA: 5-hydroxyindoleacetic acid. *n* = 24 for both genotypes. *P < 0.05, **P < 0.01, ***P < 0.001, ****P < 0.0001; Student’s *t* test or Welch’s *t* test

We detected differences in the behavioral phenotype in *Trpm1*^−/−^ mice relative to WT mice. *Trpm1* functions predominantly as a component of the retinal ON bipolar transduction cascade and its expression elsewhere in the brain is quite minor. To determine whether there are central structural changes, we compared brain regions between *Trpm1*^−/−^ and WT mice. The cerebral cortex, olfactory bulb, and pons and medulla were significantly heavier in *Trpm1* ^−/−^ mice than in WT mice at 1 month of age (Fig. 5a). In addition, the cerebral cortex, hippocampus, midbrain, and cerebellum were significantly heavier in *Trpm1*^−/−^ mice than in WT mice at 4 months of age (Fig. 5b).

We detected a subtle expression of *Trpm1* mRNA throughout the WT mouse brain with the exception of the cerebellum (Fig. 5c). We also quantified levels of biogenic monoamines ex vivo, including dopamine, noradrenaline, serotonin, and their major metabolites using HPLC-ECD in several adult brain regions. Levels of dopamine, noradrenaline, and normetanephrine (NM) were significantly decreased in the cerebellum (Fig. 5d–f). There was no significant change in the levels of the other monoamines and their metabolites in any other brain region (Additional file 2: Fig. S2).

## Discussion

Humans with 15q13.3 microdeletion syndrome exhibit a spectrum of neurobehavioral phenotypes. Many studies suggest that OTUD7A and CHRNA7 mutations partially explain the phenotypes of 15q13.3 microdeletion syndrome. A full accounting of the microdeletion phenotypes, especially those related to hyperactivity, however, is lacking. Here, we assessed the behavior of *Trpm1*-deficient mice using a comprehensive behavioral test battery. Our data revealed abnormal behaviors in *Trpm1*-deficient mice, including reduced anxiety-like behavior, abnormal social interactions, attenuated fear and spatial memories, and the most prominent phenotype of *Trpm1* mutant mice, hyperlocomotor activity (Fig. 1–4). The lack of a significant reduction of corticosterone, which is related to anxiety-like behavior, suggests that the hyperactivity observed in *Trpm1*^−/−^ mice simulates reduced anxiety in our tests (Additional file 1: Fig. S1O), and underlies or contributes to other phenotypes of *Trpm1*^−/−^ mice.

Hyperactivity is one of the features of ADHD, and humans with 15q13.3 deletion and a relative lack of expression of genes including TRPM1, exhibit ADHD behavior [30–37]. We examined the effect of MPH, a common first-line for treatment for ADHD in humans [26]. MPH significantly increased the locomotor activity of *Trpm1*^−/−^ mice (Fig. 2i). Intriguingly, MPH-like compounds are ineffective in approximately 35% of patients with ADHD [38, 39]. Several mouse models of hyperactivity are also insensitive to MPH. The ADHD-like hyperactivity of Ndrg2-deficient mice is also not rescued by MPH [40]. *Shank2* and *Fmr1* mutant mouse models of autism display hyperactivity that is increased by the administration of MPH [41, 42]. Relevant to the effect of MPH in *Shank2-* and *Fmr1*-deficient mice, hyperactivity of *Trpm1*-deficient mice may not be related to ADHD, but instead autism which is also one of the phenotypes of 15q13.3 microdeletion syndrome (Additional file 3: Table S1) [11, 43].

In the present study, *Trpm1*^−/−^  mice displayed prominent locomotor activities (Figs. 1a, 2e) that are not observed in *mGluR6*^−/−^ mice [44]. Both mouse strains lack a functional ON visual transduction pathway and a b-wave in electroretinograms [3, 45], as well as no ON response [1, 15, 16]. Additional evidence for visual impairment in *Trpm1*^−/−^ mice comes from measurements of the spatial frequency and contrast sensitivity thresholds of the optokinetic response. Thresholds were reduced by approximately 10% and 30%, respectively, compared with WT mice [46]. While both *mGluR6-* and *Trpm1-*deficient mice lack ON BC responses, *Trpm1*^−/−^ mice showed spontaneous oscillatory firing in the RGCs, the retinal output cells [17]. An attractive idea is that these retinal oscillations might be communicated along the optic nerve to higher visual centers, resulting in hyperlocomotion in *Trpm1*^−/−^ mice.

Visual impairment can lead to several behavioral alterations in humans and mice, such as enhanced auditory, haptic, and pain sensitivities [47–56], and structural changes in the visually deprived cortex as well as in other areas [50, 57, 58]. Moreover, the visual cortex receives feedback projections from auditory and somatosensory cortices and from motor and multisensory cortices [49, 59–63]. *Trpm1*^−/−^ mice did not show hypersensitivity to sensory stimuli, at least with regard to thermal perception and auditory responses (Fig. 1b–d). Thus, it is unlikely that the behavioral changes in *Trpm1*^−/−^ mice are secondary to changes in non-visual sensory perception. We cannot, however, exclude the possibility that visual impairment in *Trpm1*^−/−^ mice somehow contributes to the emotional phenotypes in the mice. Some visionally impaired mice show altered anxiety-like behaviors. For example, *rd8* mice, in which photoreceptors have degenerated and vision is impaired, show hypolocomotor activity and increased anxiety-like behavior [64, 65], an emotional phenotype opposite that observed in *Trpm1*^−/−^ mice.

Another possible explanation for the behavioral phenotypes in *Trpm1*^−/−^ mice is that deficiency of *Trpm1* expression in the brain leads to a neurochemical attenuation in brain function that may cause the behavioral phenotypes. TRPM1 is expressed in the retina and skin in humans [66–68], and a short form of TRPM1, which does not have channel function, is expressed in embryonic retinal pigment epithelial and skin in mice [1, 2]. Thus, there is a precedent for the expression of TRPM1 outside of the retina, including alternative splice forms. We analyzed the expression of *Trpm1* in the brain and detected a faint expression by qPCR throughout most of the brain with the exception of the cerebellum (Fig. 5c). Hence, *Trpm1* may be expressed in some parts of the brain and the presence or lack of *Trpm1* in a particular region may affect behavior. The lack of an overlap between the *Trpm1* expression pattern and the change in the monoamine distribution in the brain (Fig. 5d–f) is consistent with the idea that *Trpm1* is expressed in monoaminergic neurons that project to the cerebellum. A link between TRPM1 and brain function was previously suggested by the demonstration that capsaicin-induced activation of TRPM1 channels contributes to the induction of long-term depression in the lateral amygdala, which is specifically mediated by group I mGluRs and interactions with another member of the TRP family, TRPC5 [69]. Deficiency of *Trpm1* expression in the brain, including the amygdala, may lead to a neurochemical attenuation in brain function, thereby causing that may cause behavioral phenotypes in *Trpm1-*deficient mice similar to those demonstrated here.

In summary, our results are consistent with the idea that spontaneous oscillatory firing in the retina may be transmitted to the higher visual system through the optic nerve and more central projections during development and later, and as a result may modify the function and structure of the brain leading to the observed behavioral changes. An alternative, but not mutually exclusive, possibility is that the lack of expression of *Trpm1* in the brain changes the distribution of biogenic monoamines and behaviors in *Trpm1*^−/−^ mice. Irrespective of the mechanism, this is the first report to implicate TRPM1 loss in 15q13.3 microdeletion syndrome. Further experiments are needed to determine if retinal dysfunction causes brain alterations, or whether TRPM1 makes specific contributions in certain brain regions.

## Supplementary Information

**Additional file 1: Figure S1.** Behavioral and physiological characteristics of *Trpm1*^−/−^ mice. (**A**–**E**) General health and neurological screen; (**A**) body weight, (**B**) body temperature, (**C**) grip strength, (**D**) wire hang test, (**E**) latency to fall in the rotarod test. *n* = 4 for both genotypes (**A**–**D**), *n* = 23 for both genotypes (**E**). (**F**–**K**) Gait analysis of front and hind paws; (**F**) stride duration, (**G**) stance duration, (**H**) stride length, (**I**) stance width, (**J**) step angle, (**K**) paw angle. *n* = 24 Trpm1^−/−^, *n* = 23 WT. (**L**, **M**) Porsolt forced swimming test; (**L**) distance traveled, and (**M**) proportion of time spent immobile in each 1-min period. *n* = 24 for both genotypes. (**N**) Percentage of time spent immobile in each 1-min period in the tail suspension test. *n* = 24 for both genotypes. (**O**) Serum corticosterone was measured at 4 months of age. *n* = 4 for both genotypes. (**P**) Social activity averaging 3 days in home cage test. *n* = 22 Trpm1^−/−^, *n* = 21 WT. *P < 0.05, **P < 0.01, ****P < 0.0001; 2-way ANOVA followed by Tukey’s multi comparison test. (**A**, **C**), Student’s t test (**B**, **F**–**K**, **O**), Welch’s t test (**D**) or repeated measures 2-way ANOVA (**E**, **L**–**N**, **P**).

**Additional file 2: Figure S2.** Normal biomonoamine levels in the brains (except the cerebellum) of *Trpm1*^−/−^ mice. Quantification of monoamine neurotransmitters in brain regions except the cerebellum at 4 months old. *n* = 24 for both genotypes. No significant changes; Student’s t test or Welch’s *t* test.

**Additional file 3: Table S1.** 15q13.3 microdeletion syndrome and corresponding mutant mice. –: not assessed, n.s.: no significant difference, M: male, F: female, Ref: references.

## Data Availability

The datasets in the current study are available in the [Mouse Phenotype Database] repository, [http://www.mouse-phenotype.org/].

## Abbreviations

TRPM1: Transient receptor potential cation channel subfamily M member 1
mGluR6: Metabotropic glutamate receptor 6
BC: Bipolar cell
MTMR10: Myotubularin-related protein 10
FAN1: Fanconi-associated nuclease 1
KLF13: Kruppel-like factor 13
MIR211: MicroRNA 211
OTUD7A: OTU deubiquitinase 7A
CHRNA7: Cholinergic receptor nicotinic alpha 7 subunit
ADHD: Attention-deficit hyperactivity disorder
RGC: Retinal ganglion cell
WT: Wild-type
CS: Conditioned stimulus
US: Unconditioned stimulus
MPH: Methylphenidate
HPLC: High-performance liquid chromatography
ECD: Electrochemical detector
qPCR: Quantitative polymerase chain reaction
ELISA: Enzyme-linked immunosorbent assay
ANOVA: Analysis of variance
DOPAC: ﻿3,4-Dihydroxyphenylacetic acid
3-MT: 3-Methoxytyramine
HVA: Homovanillic acid
Ad: Adrenaline
MHPG: 3-Methoxy-4-hydroxyphenylglycol
5-HIAA: 5-Hydroxyindoleacetic acid
