# Supplementary material for: Mice with mutations in Trpm1, a gene in the locus of 15q13.3 microdeletion syndrome, display pronounced hyperactivity and decreased anxiety-like behavior
Source: Mol Brain. 2021 Mar 30;14:61. doi: 10.1186/s13041-021-00749-y (PMC8008678; doi:10.1186/s13041-021-00749-y)
Supplement: Supplementary file 1 — Additional file 1: Figure S1. Behavioral and physiological characteristics of Trpm1−/− mice. (A–E) General health and neurological screen; (A) body weight, (B) body temperature, (C) grip strength, (D) wire hang test, (E) latency to fall in the rotarod test. n = 4 for both genotypes (A–D), n = 23 for both genotypes (E). (F–K) Gait analysis of front and hind paws; (F) stride duration, (G) stance duration, (H) stride length, (I) stance width, (J) step angle, (K) paw angle. n = 24 Trpm1−/−, n = 23 WT. (L, M) Porsolt forced swimming test; (L) distance traveled, and (M) proportion of time spent immobile in each 1-min period. n = 24 for both genotypes. (N) Percentage of time spent immobile in each 1-min period in the tail suspension test. n = 24 for both genotypes. (O) Serum corticosterone was measured at 4 months of age. n = 4 for both genotypes. (P) Social activity averaging 3 days in home cage test. n = 22 Trpm1−/−, n = 21 WT. *P < 0.05, **P < 0.01, ****P < 0.0001; 2-way ANOVA followed by Tukey’s multi comparison test. (A, C), Student’s t test (B, F–K, O), Welch’s t test (D) or repeated measures 2-way ANOVA (E, L–N, P). [file 13041_2021_749_MOESM1_ESM.pdf]

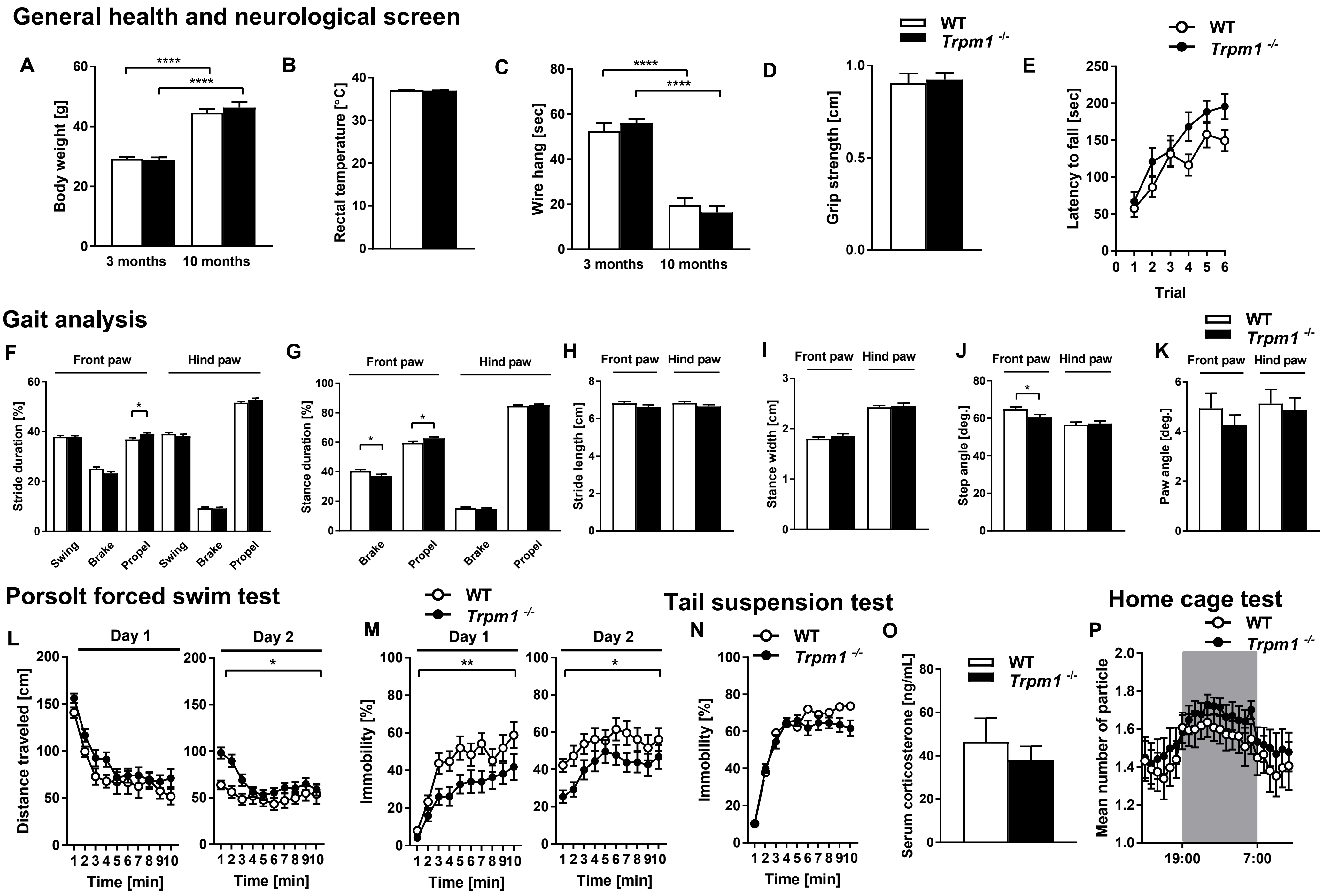
General health and neurological screen
WT
Trpm1 -/-
A
Body weight [g]
****
****
3 months
10 months
B
Rectal temperature [°C]
C
Wire hang [sec]
****
****
3 months
10 months
D
Grip strength [cm]
E
Latency to fall [sec]
Trial
Gait analysis
F
Front paw
Hind paw
Stride duration [%]
Swing
Brake
Propel
G
Stance duration [%]
H
Stride length [cm]
I
Stance width [cm]
J
Step angle [deg.]
K
Paw angle [deg.]
Porsolt forced swim test
L
Day 1
Day 2
Distance traveled [cm]
Time [min]
M
Immobility [%]
Tail suspension test
N
O
Serum corticosterone [ng/mL]
Home cage test
P
Mean number of particle
19:00
7:00
