# Supplementary figures and images for: Mice with mutations in Trpm1, a gene in the locus of 15q13.3 microdeletion syndrome, display pronounced hyperactivity and decreased anxiety-like behavior
Source: Mol Brain. 2021 Mar 30;14:61. doi: 10.1186/s13041-021-00749-y (PMC8008678; doi:10.1186/s13041-021-00749-y)

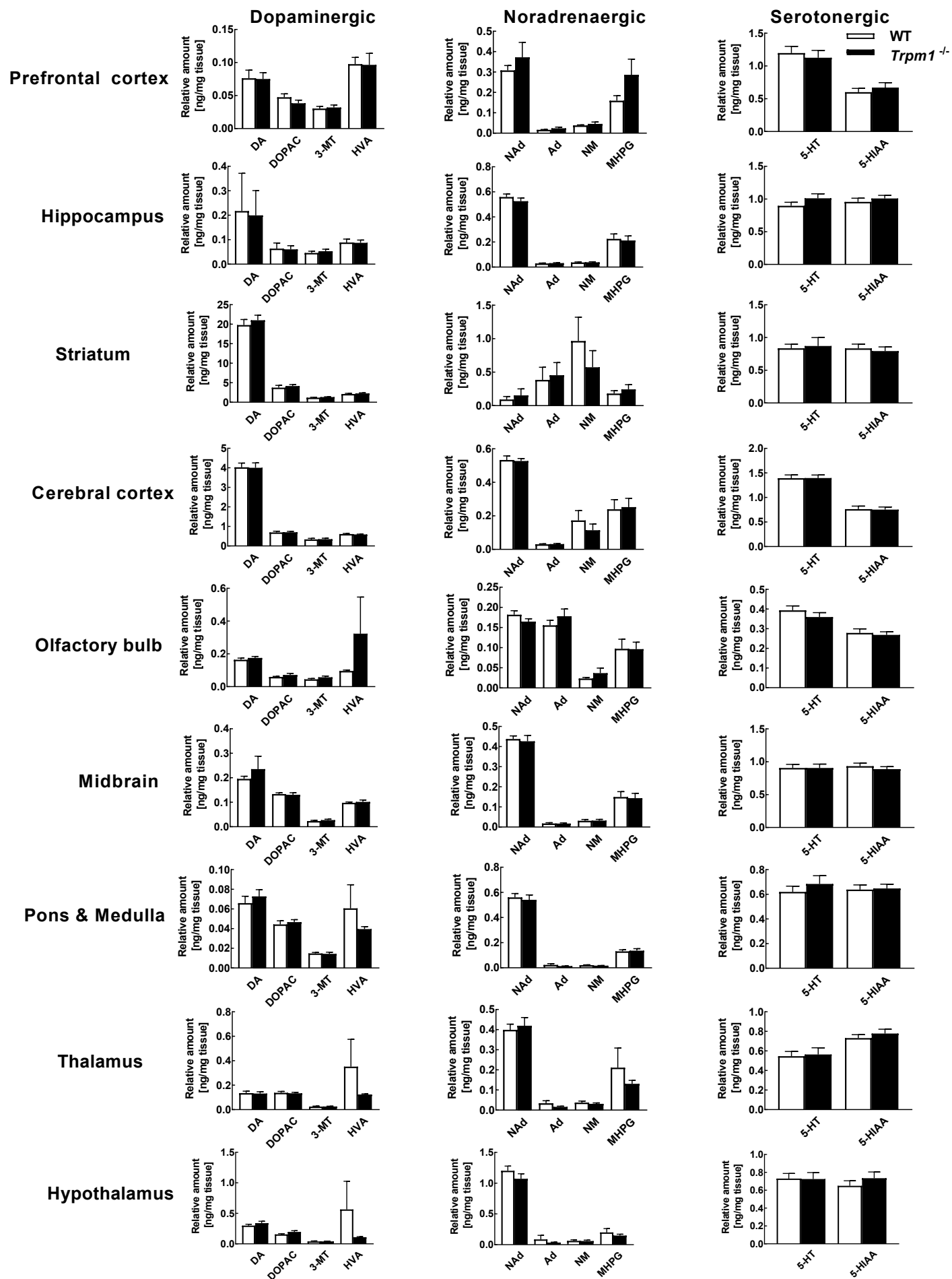

Supplement: Supplementary file 2 — Additional file 2: Figure S2. Normal biomonoamine levels in the brains (except the cerebellum) of Trpm1−/− mice. Quantification of monoamine neurotransmitters in brain regions except the cerebellum at 4 months old. n = 24 for both genotypes. No significant changes; Student’s t test or Welch’s t test. [file 13041_2021_749_MOESM2_ESM.pdf]
