# Supplementary material for: Mice with mutations in Trpm1, a gene in the locus of 15q13.3 microdeletion syndrome, display pronounced hyperactivity and decreased anxiety-like behavior
Source: Mol Brain. 2021 Mar 30;14:61. doi: 10.1186/s13041-021-00749-y (PMC8008678; doi:10.1186/s13041-021-00749-y)
Supplement: Supplementary file 3 — Additional file 3: Table S1. 15q13.3 microdeletion syndrome and corresponding mutant mice. –: not assessed, n.s.: no significant difference, M: male, F: female, Ref: references. [file 13041_2021_749_MOESM3_ESM.pdf]

Phenotype of 15q13.3 microdeletion patients	Behavioral test in male mice	Df(h15q13) −/− see Ref. 49	Trpm1 −/− Current study	Otud7a −/− see Ref. 7	Chrna7 −/− see Ref. 6
Developmental delay	Developmental milestones	–	–	Impaired (M+F)	–
Language impairment	Ultrasonic vocalization	Impaired (M+F)	–	Impaired (M+F)	–
Intellectual disability	Conditioned fear	Impaired cued (F)	Impaired context and cued (M)	Increased cued (M)	–
	Novel object recognition	–	–	n.s. (M)	–
	T-maze	–	n.s. (M)	–	–
	Morris water maze	Impaired (M)	–	–	–
Motor deficits	Rotarod	–	n.s. (M)	Impaired (M)	–
Schizophrenia	Prepulse inhibition	Impaired (M)	Impaired at 110 dB startle (M)	n.s. (M)	n.s. (M)
Autism (social interaction deficits)	Three-chamber test	Not impaired (F)	n.s. (M)	Not impaired (M+F)	n.s. (M)
	Reciprocal social interaction	–	Increased (M)	–	n.s. (M)
	Partition test	–	–	n.s. (M)	n.s. (M)
	Nest building	Impaired (F)	–	n.s. (M)	–
Autism (repetitve behaviors)	Self-grooming	Normal (M)	Increased (M)	n.s. (M)	n.s. (M)
	Holeboard exploration	–	–	n.s. (M)	n.s. (M)
	Marble burying	–	–	–	Increased (M)
Hypotonia	Grip strength	Decreased (M)	n.s. (M)	n.s. (M)	–
Anxiety	Elevated plus maze	–	Decreased (M)	n.s. (M+F)	n.s. (M)
	Light-dark box exploration	–	Decreased (M)	n.s. (M)	–
Hyperactivity	Open field test	n.s. (M)	Increased (M)	n.s. (M)	n.s. (M)
Depression	Forced swimming test	–	n.s. (M)	n.s. (M)	n.s. (M)
	Tail suspension test	–	n.s. (M)	–	n.s. (M)
Aggressive	Tube test	–	–	–	n.s. (M)
